# Stress Analysis and Stiffness Degradation of Open Cracks Composite Laminates Subjected to External Loads

**DOI:** 10.3390/biomimetics10030177

**Published:** 2025-03-12

**Authors:** Zhicheng Huang, Shengyun Su, Xingguo Wang, Fulei Chu

**Affiliations:** 1 School of Mechanical and Electronic Engineering, Jingdezhen Ceramic University, Jingdezhen 333403, China; 2 School of Mechanical Engineering, Tsinghua University, Beijing 100084, China

**Keywords:** biomimetic composite laminates, composite symmetric laminates, open cracks, the structural and material parameters, stress analysis, stiffness degradation

## Abstract

Composite laminated structures have extensive applications in the field of bionic engineering. Proficient comprehension of the mechanical properties of these structures is instrumental in the advancement of bionic composite materials. The objective of this study is to investigate the stress distribution and degradation of stiffness in composite laminates exhibiting open smooth surface cracks under varying external loads and structural parameters. Utilizing the general series function of the laminate’s axial stress, the general expression for the stress components of the damaged laminate is derived by integrating the equilibrium differential equation, boundary conditions, and stress continuity conditions. The influence of fiber orientation and material properties on the stress distribution within each layer of symmetric composite laminates was examined. Thereafter, the reduction in cross-layer shear modulus was assessed by employing the principle of complementary energy minimization. The impact of structural parameters on shear modulus reduction was explored. The findings indicate that structural and material parameters of symmetric laminates featuring transverse matrix cracks exert a notable influence on the stress distribution and degradation of stiffness within each layer, imparting practical significance to the research outcomes in engineering applications.

## 1. Introduction

Bionics, also referred to as biomimicry or heuristics, entails the study of resolving artificial intelligence and technological challenges by emulating the characteristics and processes of biological systems [1,2,3]. This interdisciplinary field integrates knowledge and methodologies from various disciplines, including biology, physics, chemistry, mathematics, and computer science. The research in bionics is comprehensive, encompassing areas such as biomimetic robotics, biomimetic materials, and biomimetic algorithms. It represents the cutting edge in exploring the integration of natural intelligence with artificial constructs, propelling materials science toward new frontiers. In this domain, there is a growing interest in composite laminated structures due to their novel design concepts and practical utility. Composite sandwich structures, in particular, find diverse applications in bionic engineering, such as the development of bionic shrimp shell structures, bionic glass sandwich panels, and impact-resistant bionic mantis shrimp sandwich boards. However, the use of composite materials may result in internal cracks and damage within the laminated structures, which can severely compromise their mechanical properties. Consequently, numerous scholars have conducted extensive research on these layered structures. In general angle-laid laminates, the initial form of damage incurred under external loading is the occurrence of transverse matrix cracks in the 90° layer [4,5,6,7,8,9]. Such cracks are likely to decrease the stiffness of the laminates and pose a significant damage potential. Hajikazemi [10] proposed a more accurate method to calculate the effective thermal expansion coefficient of the cracked laminates and combined with the allowable stress field, the degradation of the thermoelastic constant of the laminates with transverse cracks could be predicted more accurately and then compared with the calculated results of various models. Finally, it is found that the calculated results of this method are more accurate than those of the shear hysteresis model. In the stress-based variational model established by Huang [11], the normal stress of two adjacent layers of the crack layer is introduced into the statically indeterminate boundary problem. Then, by using the principle of minimum complementary energy, the stress components of each layer can be derived by the variational method and solved by the governing equation. Then, according to the classical elastic theory of laminates, Huang [12] proposed that there are two types of transverse matrix cracks in angle-laid laminates, namely, open cracks and closed cracks. After variational analysis of these two kinds of cracks, it is found that there are great differences in stress distribution and effective mechanical properties between the open crack and the closed crack laminates. Huang [13] found that most current studies have ignored the influence of fiber bridging resistance between irregularly cracked surfaces on the effective mechanical properties of damaged laminates, and their research results are limited to situations where the cracked surface is smooth. In 2010, Cortes and Barbero [14] applied their method to any stacking order of symmetric laminates with diagonal cross-matrix cracks and matrix cracks in one or two directions, showing good prediction ability for crack initiation and evolution in different laminates. In 2014, Carraro [15] studied the stiffness degradation of multidirectional symmetric laminates under the action of off-axis cracks. A new analytical model is proposed, which can be used to predict the elastic properties of multidirectional symmetric laminates with arbitrary layering and one or more layers of cracks. The model is based on the so-called “optimal” shear hysteresis analysis of single-layer (symmetrically) cracked laminates and an analytical procedure that explains the presence of multilayer cracks while taking into account their interactions. The predicted results are in good agreement with the experimental data in the literature. The calculation results also show that considering the interaction of cracks between different layers is the basis for estimating stiffness changes reliably. Liang Hanbing [16] set the interlaminar shear stress in the form of a general function and obtained the stress component expression of the opening crack from the equilibrium equation and boundary conditions. On this basis, the governing equations of the opening crack were obtained by using the principle of minimum complementary energy, and then the stress distribution of the symmetric laminates of composite materials was finally obtained by analytical analysis and solution. The effect of fiber bridging on the shear modulus of composite laminates is analyzed, and the effect of bridging factors on the effective shear modulus is calculated. Huang [17] then obtained an exact solution of the stress in the cracked laminate, taking into account the series expansion form of the sine function of the shear stress between the 90° layer and the 0° layer. Meanwhile, the results of the interlayer stress show that the delamination occurs near the splitting position. Duan and Gan [18] proposed a semi-analytic finite element method to simulate composite laminates with arbitrary fiber orientation and anisotropic material properties in each layer. Hajikazemi and Sadr [19] established a variational model to analyze the stress field of symmetrically fractured laminates under general loads in the plane. Thus, the applicability of variational methods in considering multilayered symmetric laminates with arbitrary superimposed sequences is fundamentally extended. They compare the results of stress distribution in the literature for the case of orthogonal laminates. In this paper, Hajikazemmi [10] tries the energy variational principle for crack analysis of symmetric laminates with arbitrary lamination order under general plane loading. Some scholars, such as Zhang [20,21,22], assumed that the shear stress in laminates is linear through the entire thickness layer in the 90° layer, while the shear stress is equal to zero in the 0° layer. In view of the energy variational principle, Nairn [23], Varna and Berglund [24,25], and Berglund and Varna [26] further refined the model by assuming that in the 90° layer, the shear stress $\tau_{zx}$ is a linear function of z and the stress $\sigma_{z}$ is a quadratic function of z. The 0° layer may have a non-uniform stress distribution described by an exponential function with unknown shape parameters. The problem is reduced to a fourth-order differential equation with constant coefficients to find the minimum value of the additional energy. The equation and its solution contain an unknown shape parameter, which is calculated in the subsequent additional energy minimization. Berglund’s improvement introduced the statically indeterminate boundary problem. In 2014, Carraro [15] used an analytical method to provide a computational model for the elastic properties of any number of layered symmetric laminates, taking into account their interactions. For this reason, the two-dimensional optimal shear hysteresis analysis of laminates with single cracks was carried out for the first time. Gabbert et al. [7] studied unidirectional fiber composites with unsatisfactory interface conditions between reinforcement and filler. The microstructure is periodic and the phase is isotropic. The periodicity of the microstructure appears as a parallelogram. Using the concept of representative volume element (RVE), a finite element model of fiber distribution and incomplete contact conditions between phase interfaces is established. June M and Kayran A [27] conducted a finite element analysis of the geometric nonlinear progressive failure of perforated composite laminates under both in-plane and out-of-plane composite loads. Different failure criteria and material property degradation schemes based on layers and components were written, and a progressive failure analysis program was developed. Based on the finite element method, Vejen and Pyrz [28] studied the transverse crack propagation of long fiber composites. Three criteria of pure matrix growth, fiber/matrix interface growth, and fiber/matrix crack bending outside the interface are realized, and a crack propagation module of a software package is formed. The numerical crack path is compared with the experimental crack path. Finally, the crack propagation module is used to construct an example to obtain information about the influence of various fiber distributions on the crack propagation process. Ravi Joshi [29] applies evenly distributed transverse loads to the antisymmetric and symmetric composite laminates and conducts failure analysis for each layer in turn. Hashin [30] analyzed 90 of them using a variational method. The tensile and shear loads of the laminates with transverse matrix cracks are contained in the laminates. The allowable stress system satisfying the equilibrium condition and all boundary and interface conditions is constructed, and the best approximation is found by using the principle of minimum complementary energy. Xu Dong [31] not only considers three ideal crack conditions of smooth plane opening crack, smooth plane closing crack, and fully meshing opening crack, but also analyzes two conditions of non-ideal crack, that is, non-ideal opening crack and non-ideal closing crack. The study not only verifies the symmetry of the flexibility element and the equality of the change rate of the shear flexibility element under closed conditions but also shows that the non-ideal degree of crack has a great influence on the effective coupling coefficient and the effective shear flexibility. Giannadakis [32] applies a more detailed and accurate method to estimate stress using shape function dependencies with parameters minimized. Then, the results of coenergy are compared with the corresponding results in the literature, and finally the expression of shear modulus degradation is derived. An extensive review of the literature reveals that previous research has concentrated primarily on stress distribution within individual composites or solely on the 90° cracked layer, with a specific focus on the alterations in positive (tangential) stress distribution across the plane and between layers at varying crack densities (CD). Here, CD refers to the number of cracks per unit length. Limited studies have examined the influence of fiber orientation—defined as the angle between fiber direction and the laminate’s x-axis—on the interlaminar stress distribution’s variability, as well as the impact of diverse material characteristics on the internal stress distribution in composite laminates with exposed cracks. This paper introduces a stress distribution analysis model for fiber-reinforced composite laminates featuring smooth surface cracks, characterized by the absence of contact and shear force at the crack interface. An integral contribution of this study is the derivation of a general expression for the internal stress components in interlaminar plates, achieved through the employment of the variational method and energy principles. These expressions satisfy the criteria of elastomer equilibrium differential equations, stress boundary conditions, and stress continuity. Initially, the paper examines the impact of fiber orientation on the stress distribution within a single composite layer with smooth cracks subjected to two distinct external loads—tensile and shear—and elucidates the mechanisms behind in-plane tangential and interlaminar shear stress distribution. Subsequent sections provide an in-depth analysis of various composite materials, including glass/epoxy 1, glass/epoxy 2, and carbon fiber/epoxy, with a focus on stress distribution under different material properties. The paper concludes with an investigation into the influence of matrix crack propagation on the shear modulus of composite laminates, utilizing the principle of complementary energy minimization and accounting for different structural parameters, such as the number of 0° single layers, thickness, and the thickness of crack layers. These investigations comprehensively elucidate the effects of fiber orientation and material properties on the stress distribution and shear modulus following matrix crack diffusion. This research advances our understanding of how crack diffusion affects the mechanical properties of composite laminates, fostering the development of mechanics and stiffness degradation theories for these materials, and providing a robust theoretical foundation for the engineering applications of fiber-reinforced composites.

## 2. Boundary Conditions and Stress Components

### 2.1. Element Volume Model

**Figure 1:**
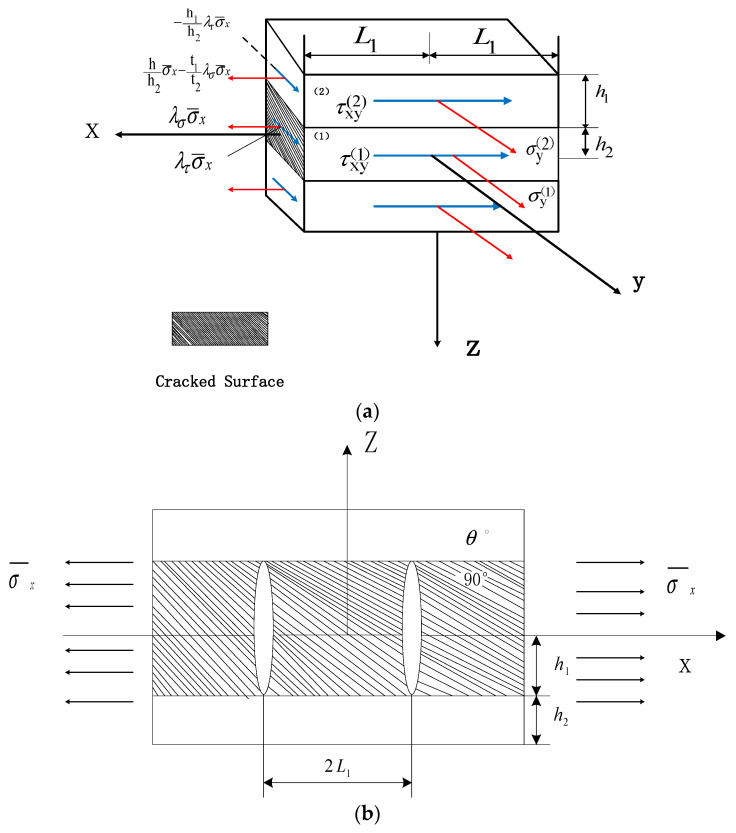
(**a**) Open Cracked elements under in-plane shear loading. (**b**) Crack model of laminates.

The early experimental results [4,5,6,7,8,9] demonstrate that as the crack density increases, transverse cracks within the 90° layer of the matrix-cracked laminates are uniformly distributed along the length of the plate, extending across the full width and throughout the entire thickness. Tensile stress ${\bar{\sigma }}_{x}$ is exerted on the composite laminates ${\left[\theta_{m}/90_{n}\right]}_{s}$ with laterally cracked matrix. As shown in Figure 1a,b, the thickness of the 90° layer and $\theta^{{}^{\circ}}$ layer is $h_{1}=nt_{0}$. $t_{0}$ is the thickness of the single-layer plate. Subscript 1 represents the 90° layer, and subscript 2 represents $\theta^{{}^{\circ}}$ layer. The distance between the two cracks is $2L_{1}$. Assuming that the cracks are equally spaced, the crack density is CD = $1/2L_{1}$. The total thickness of the laminate is $2h=2h_{1}+2h_{2}$. The whole layer is laid in accordance with $\theta^{{}^{\circ}}$, 90°, $\theta^{{}^{\circ}}$, and the structure of the laminate has upper and lower symmetry. Therefore, only the upper part of the volume element needs to be studied.

### 2.2. No Thickness Spring Model

Based on the actual defects present in the material, the non-ideal matrix crack is conceptualized as a defect interface. Taking into account the continuity of stress across the defect and the discontinuity of displacement, the theoretical model of spring without thickness is established.

(1) $$\sigma_{x}^{\left(1\right)}{|}_{x=L_{1}}=\lambda_{\sigma }{\bar{\sigma }}_{x}=K_{1}\left[\left[\alpha \right]\right]$$

(2) $$\tau_{xy}^{\left(1\right)}{|}_{x=L_{1}}=\lambda_{\tau }{\bar{\sigma }}_{x}=K_{2}\left[\left[\beta \right]\right],$$

where $K_{1}$ and $K_{2}$ represent imperfect interface parameters, and expressed in units of Pa/m. $\left[\left[\alpha \right]\right]=\alpha^{+}-\alpha^{-}$ and $\left[\left[\beta \right]\right]=\beta^{+}-\beta^{-}$ denote the differences in normal and tangential displacements, respectively, between the two opposite surfaces of the defect. The values of the two independent imperfect interface parameters directly indicate the extent of ideal versus non-ideal defects. ① When $K_{1}=0$, $K_{2}=0$, the normal and tangential displacements are discontinuous, and the corresponding normal and tangential stresses are zero, indicative of a smooth plane opening crack. ② When $K_{1}\to \infty$, $K_{2}=0$, the normal displacement is continuous, the normal displacement remains continuous, the normal stress is non-zero, the tangential displacement is discontinuous, the tangential stress is zero, and a smooth plane closed crack can be characterized. ③ When $K_{1}\to 0$, $K_{2}\to \infty$, the normal displacement is discontinuous, the normal stress is zero, the tangential displacement is continuous, the tangential stress is non-zero, and a fully meshed opening crack can be characterized. (It should be noted that if the mesh represents a closed crack, the normal and tangential displacements are continuous, and the corresponding normal and tangential stresses are non-zero, which may indicate no damage). ④ When $K_{1}\to \infty$, $K_{2}\to \infty$, the normal and tangential displacements are continuous, and the corresponding normal and tangential stresses are non-zero, indicative of a non-destructive injury scenario.

### 2.3. Boundary Conditions

For the statically indeterminate boundary, two undetermined zonal loads are introduced in accordance with tensile loading. Subsequently, the directional stress and tangential stress within regions (1) and (2) at the RVE boundary, under various loading conditions, must satisfy the following criteria:

For tensile loading, $\lambda_{\sigma }{\bar{\sigma }}_{x}$ and $\lambda_{\tau }{\bar{\sigma }}_{x}$ are introduced:

(3) $$\left\{\begin{array}{l} h_{1}\sigma_{x}^{\left(1\right)}{|}_{x=\pm L_{1}}+h_{2}\sigma_{x}^{\left(2\right)}{|}_{x=\pm L_{1}}=h\bar{\sigma_{x}}\\ h_{1}\tau_{xy}^{\left(1\right)}{|}_{x=\pm L_{1}}+h_{2}\tau_{xy}^{\left(2\right)}{|}_{x=\pm L_{1}}=0\\ h_{1}\sigma_{x}^{\left(1\right)}{|}_{x=\pm \infty }+h_{2}\sigma_{x}^{\left(2\right)}{|}_{x=\pm \infty }=0 \end{array}\right..$$

Therefore, the boundary conditions are expressed as follows [31]:

(4) $$\begin{array}{l} \sigma_{x}^{\left(1\right)}{|}_{x=\pm L_{1}}=\lambda_{\sigma }\bar{\sigma_{x}}\;,\;\tau_{\mathrm{xy}}^{\left(1\right)}{|}_{x=\pm L_{1}}=\lambda_{\tau }{\bar{\sigma }}_{x}\\ \sigma_{x}^{\left(2\right)}{|}_{x=\pm L_{1}}=\frac{h}{t_{2}}\bar{\sigma_{x}}-\frac{h_{1}}{h_{2}}\lambda_{\sigma }\bar{\sigma_{x}}\;,\;\tau_{\mathrm{xy}}^{\left(2\right)}{|}_{x=\pm L_{1}}=-\frac{h_{1}}{h_{2}}\lambda_{\tau }{\bar{\sigma }}_{x} \end{array}.$$

In symmetrically laminated plates, the external shear stress at the middle surface (Z = 0) of layer (1) is zero, viz:

(5) $$\tau_{\mathrm{zx}}^{\left(1\right)}{|}_{z=0}=\tau_{\mathrm{zy}}^{\left(1\right)}{|}_{z=0}=0.$$

On the surface of the laminate, at the top of layer (2) (Z = *h*), the surface shear stress and normal stress are 0, that is,

(6) $$\sigma_{z}^{\left(2\right)}{|}_{z=h}=0,\tau_{\mathrm{xz}}^{\left(2\right)}{|}_{z=h}=\tau_{\mathrm{yz}}^{\left(2\right)}{|}_{z=h}=0.$$

### 2.4. The Stress Components Within Each Layer

The load is uniformly distributed along the Y-axis. Hence, the stress component may be presumed to be a function of the variables x and z. This allows for the additional simplification that the out-of-plane shear stress is linearly dependent on the thickness of each layer, while the in-plane stress within each layer is independent of z. Consequently, under tensile loading, the interlaminar stress between region (1) and the adjacent region (2) is represented by Equation (7) as follows:

(7) $$\tau_{\mathrm{zx}}^{\left(12\right)}={\bar{\sigma }}_{x}\varphi^{\prime }(\varsigma )\;\;\;\;\;\;\tau_{\mathrm{zy}}^{\left(12\right)}={\bar{\sigma }}_{x}\psi^{\prime }(\varsigma ),$$

where in the superscript (12) denotes the interface between layers (1) and (2) and $\varsigma =x/L_{1}$ is a dimensionless variable. The combined formulas from (3) to (6), along with the interlayer stress conditions, must remain continuous. These can be individually derived for the stress components within each zone under tensile loading.

For the 90° layer,

(8) $$\begin{array}{l} \sigma_{x}^{\left(1\right)}=-\frac{L_{1}}{h_{1}}{\bar{\sigma }}_{x}\varphi (\varsigma )+\lambda_{\sigma }{\bar{\sigma }}_{x},\;\sigma_{y}^{\left(1\right)}=\frac{L_{1}}{h_{1}}{\bar{\sigma }}_{x}\phi (\varsigma ),\sigma_{z}^{\left(1\right)}=\frac{1}{2}(h-\frac{z^{2}}{h_{1}})\frac{1}{L_{1}}{\bar{\sigma }}_{x}\varphi^{\prime \prime }(\varsigma )\;\\ \tau_{zx}^{\left(1\right)}=\frac{z}{h_{1}}{\bar{\sigma }}_{x}\varphi^{\prime }(\varsigma ),\tau_{zy}^{\left(1\right)}=\frac{z}{h_{1}}{\bar{\sigma }}_{x}\psi^{\prime }(\varsigma ),\tau_{\mathrm{xy}}^{\left(1\right)}=-\frac{L_{1}}{h_{1}}{\bar{\sigma }}_{x}\psi (\varsigma )+\lambda_{r}{\bar{\sigma }}_{x} \end{array}.$$

For the $\theta^{{}^{\circ}}$ layer,

(9) $$\begin{array}{l} \sigma_{x}^{\left(2\right)}=\frac{L_{1}}{h_{2}}{\bar{\sigma }}_{x}\varphi (\varsigma )+\frac{h}{h_{2}}{\bar{\sigma }}_{x},\;\sigma_{y}^{\left(2\right)}=-\frac{L_{1}}{h_{2}}{\bar{\sigma }}_{x}\phi (\varsigma ),\;\sigma_{z}^{\left(2\right)}=\frac{{\left(h-z\right)}^{2}}{2h_{2}}\frac{1}{L_{1}}{\bar{\sigma }}_{x}\varphi^{\prime \prime }(\varsigma )\\ \tau_{\mathrm{zx}}^{\left(2\right)}=\frac{h-z}{h_{2}}{\bar{\sigma }}_{x}\varphi^{\prime }(\varsigma ),\;\tau_{\mathrm{zy}}^{\left(2\right)}=\frac{h-z}{h_{2}}{\bar{\sigma }}_{x}\psi^{\prime }(\varsigma ),\;\tau_{\mathrm{xy}}^{\left(2\right)}=\frac{L_{1}}{h_{2}}{\bar{\sigma }}_{x}\psi (\varsigma )-\frac{h_{1}}{h_{2}}\lambda_{r}{\bar{\sigma }}_{x} \end{array}.$$

Taking into account the continuity of stress, Equations (8) and (9), when combined with the boundary conditions, yield the following:

(10) $$\varphi (\varsigma ){|}_{\varsigma =\pm 1}=\psi (\varsigma ){|}_{\varsigma =\pm 1}=0,\;\varphi^{\prime }(\varsigma ){|}_{\varsigma =\pm 1}=0.$$

Within the formula φ(ς), ψ(ς) and ϕ(ς) represent three independent undetermined functions that are employed to depict the interlayer stress $\lambda_{\sigma }$, and $\lambda_{\tau }$ denotes undetermined load distribution coefficients. Each of these unknown functions and coefficients can be resolved using the governing equation.

## 3. Governing Equation and Supplementary Equation

### 3.1. Establishing Governing Equations and Supplementary Equations

For open cracks:

In adherence to the principle of minimum complementary energy, the governing equations for the functions φ(ς), ψ(ς) and ϕ(ς) can be established through variational analysis and are expressed as follows:

(11) $$\delta U_{\sigma }=0,$$

where *U* is the complementary energy given by

(12) $$U=U^{1}+{\int_{-L_{1}}^{L_{1}}\int_{0}^{h_{1}}\mu }^{\left(1\right)}dzdx+\int_{-L_{1}}^{L_{1}}\int_{h_{1}}^{h}\mu^{\left(2\right)}dzdx,$$

in which

(13) $$\mu^{\left(i\right)}=\frac{1}{2}[\sigma_{x}^{\left(i\right)}\varepsilon_{x}^{\left(i\right)}+\sigma_{y}^{\left(i\right)}\varepsilon_{y}^{\left(i\right)}+\sigma_{z}^{\left(i\right)}\varepsilon_{z}^{\left(i\right)}+\tau_{zx}^{\left(i\right)}\gamma_{zx}^{\left(i\right)}+\tau_{zy}^{\left(i\right)}\gamma_{zy}^{\left(i\right)}+\tau_{xy}^{\left(i\right)}\gamma_{xy}^{\left(i\right)}]\;\;\;\;\;(i=1,2),$$

(14) $$U^{1}=\frac{1}{2k_{1}}{(\;\lambda_{\sigma }{\bar{\sigma }}_{x})}^{2}t_{1}+\frac{1}{2k_{2}}{(\;\lambda_{\tau }{\bar{\sigma }}_{x})}^{2}t_{1},$$

and $\varepsilon_{x}$, $\varepsilon_{y}$, $\varepsilon_{z}$, $\gamma_{\mathrm{zx}}$, $\gamma_{\mathrm{zy}}$ and $\gamma_{\mathrm{xy}}$ are the normal and shear strain components, respectively.

Substituting Equations (8) and (9) into Equation (13) and applying Hooke’s law and Equation (11) yield the non-zero governing equation, given by δφ, δψ and δϕ:

(15) $$\begin{array}{l} A_{11}\varphi^{\prime \prime \prime \prime }(\varsigma )+B_{11}\varphi^{\prime \prime }(\varsigma )+C_{11}\varphi (\varsigma )+B_{12}\psi^{\prime \prime }(\varsigma )\\ +C_{12}\psi (\varsigma )+B_{13}\phi^{\prime \prime }(\varsigma )+C_{13}\phi (\varsigma )=D_{1}\\ B_{21}\varphi^{\prime \prime }(\varsigma )+C_{21}\varphi (\varsigma )+B_{22}\psi^{\prime \prime }(\varsigma )+C_{22}\psi (\varsigma )+C_{23}\phi (\varsigma )=D_{2} \end{array}.$$

The coefficients ($A_{ij}$, $B_{ij}$, $C_{ij}$) and the constants $D_{i}$ are listed in Appendix A.

The governing equations for the functions φ(ς) and ψ(ς) are obtained as

(16) $$\begin{array}{l} a_{11}\varphi^{\prime \prime \prime \prime }(\varsigma )+b_{11}\varphi^{\prime \prime }(\varsigma )+c_{11}\varphi (\varsigma )+b_{12}\psi^{\prime \prime }(\varsigma )+c_{12}\psi (\varsigma )=d_{1}\\ b_{21}\varphi^{\prime \prime }(\varsigma )+c_{21}\varphi (\varsigma )+b_{22}\psi^{\prime \prime }(\varsigma )+c_{22}\psi (\varsigma )=d_{2} \end{array}.$$

The function ϕ(ς) can be obtained as

(17) $$\phi (\varsigma )=\frac{D_{3}}{C_{33}}-\frac{1}{C_{33}}[B_{31}\varphi^{\prime \prime }(\varsigma )+C_{31}\varphi (\varsigma )+C_{32}\psi (\varsigma )].$$

The coefficients ($a_{ij}$, $b_{ij}$, $c_{ij}$) and constants $d_{ij}$ are listed in Appendix B.

### 3.2. Solution to Governing and Supplementary Equations

The equations can be solved using linear differential equation theory. Formula (15) is the sum of the characteristic constants of the general solution of the corresponding homogeneous equation and the functions φ(ς) and ψ(ς) of the nonhomogeneous equations. The general solution of the homogeneous equations can be written by the root $r_{j}$ (*j* = 1, 2, …, 6) of the characteristic equation, such that

(18) $$\left|\begin{array}{l} a_{11}r^{4}+b_{11}r^{2}+c_{11}\\ b_{21}r^{2}+c_{21} \end{array}\right.\left.\begin{array}{l} b_{12}r^{2}+c_{12}\\ b_{22}r^{2}+c_{22} \end{array}\right|=0,$$

and the eigenvector $D_{\left(j\right)}={\left[\begin{array}{cc} D_{(j)1} & 1 \end{array}\right]}^{T}$ can be obtained from the following equation:

(19) $$\left(a_{11}{r_{i}}^{4}+b_{11}{r_{i}}^{2}+c_{11})D_{(j)1}=-(b_{12}r_{i}^{2}+c_{12}\right)$$

For the distinct roots in our following numerical examples, some of the characteristic roots may be the same, the general solutions to the homogeneous equations cannot be expressed as exponential function $\left(Ce^{\lambda x}\right)$ s, and the forms of the solutions need to be slightly modified to accommodate them; however, these procedures are all standard treatments in ordinary differential equations. Because the algebraic equation of order is an even order equation of r, the six roots of the equation correspond to three pairs of positive and negative roots $\pm r_{j}$ (*j* = 1, 2, 3). Therefore, the solution to a system of nonhomogeneous linear equations can be written as

(20) $$\varphi (\varsigma )=\sum_{j=1}^{3}(C_{+j}e^{r_{j}\varsigma }+C_{-j}e^{-r_{j}\varsigma })D_{(j)1}+\varphi^{*},\;\psi (\varsigma )=\sum_{j=1}^{3}(C_{+j}e^{r_{j}\varsigma }+C_{-j}e^{-r_{j}\varsigma })+\psi^{*}.$$

For different roots, where $C_{+j}$ and $C_{-j}$ (*j* = 1, 2, 3) are unknown constants $\varphi^{*}$, and $\psi^{*}$ are undetermined constants. Using the boundary conditions (10), we have $C_{+j}$ = $C_{-j}$. The solution (19) can easily be rewritten as

(21) $$\varphi (\varsigma )=\sum_{j=1}^{3}A_{j}D_{(j)1}\cosh(r_{j}\varsigma )+\varphi^{*},\psi (\varsigma )=\sum_{j=1}^{3}A_{j}\cosh(r_{j}\varsigma )+\psi^{*},$$

where $A_{j}=2C_{+j}$.

For open cracks, in order to determine the constants $A_{j}$ (j = 1, 2, 3), particular solutions $\varphi^{*}$ and $\psi^{*}$ can be obtained by combining the following homogeneous equations:

(22) $$\begin{array}{l} \varphi (1)=\sum_{j=1}^{3}A_{j}D_{(j)1}\cosh(r_{j})+\varphi^{*}=0,\psi (1)=\sum_{j=1}^{3}A_{j}\cosh(r_{j})+\psi^{*}=0\\ \varphi^{\prime }(1)=\sum_{j=1}^{3}A_{j}D_{(j)1}r_{j}\sinh(r_{j})=0,c_{11}\varphi^{*}+c_{12}\psi^{*}+\wedge_{1}\lambda_{\sigma }+\wedge_{2}\lambda_{\tau }=d_{1},\\ c_{21}\varphi^{*}+c_{22}\psi^{*}+\wedge_{3}\lambda_{\sigma }+\wedge_{4}\lambda_{\tau }=d_{2} \end{array}.$$

## 4. Stiffness Degradation Research

This section investigates the reduction in the shear modulus of orthogonal laminates with matrix cracks, utilizing a theoretical model based on the minimization of complementary energy. Variations in the shear modulus of the laminates are examined by manipulating the structure and material parameters. As per the classical lamination theory (CLT), the following is observed:

(23) $$G_{xy0}=\frac{1}{h}(G_{xy}^{\theta }h_{1}+G_{12}h_{2}).$$

Equation (23) $G_{xy}^{\theta }$ represents the shear modulus of $\theta^{{}^{\circ}}$ layer, whereas $G_{12}$ denotes the shear modulus of the unidirectional (UD) composite. The laminate is assumed to be subjected to the average in-plane shear stress, denoted as $\tau_{0}$. The shear strain of the undamaged laminate, denoted as $\gamma_{xy0}$, and the shear stresses within the laminate, denoted as $\tau_{0}^{90}$, and $\tau_{0}^{\theta }$ are as follows:

(24) $$\begin{array}{l} \gamma_{xy0}=\tau_{0}/G_{xy0}\\ \tau_{0}^{90}=G_{12}\gamma_{xy0}\\ \tau_{0}^{\theta }=G_{xy}^{\theta }\gamma_{xy0} \end{array}.$$

### 4.1. Assumed Stress Distributions

Two unknown stress disturbance functions, denoted as Φ(x) and $\Phi_{1}(x)$, are employed to characterize the shear stress distribution in the x-direction, and the following expression for shear stress is utilized:

(25) $$\begin{array}{l} \sigma_{xy}^{90}=\tau_{0}^{90}\cdot (1-\Phi (x))\\ \sigma_{xy}^{\theta }=\tau_{0}^{\theta }\cdot (1-h_{1}\cdot \Phi (x)\cdot f^{\prime }(z)) \end{array}.$$

The shear stress within the 90° layer is required to satisfy the condition of zero traction on the crack surface:

(26) $$\sigma_{xy}^{90}(x=\pm l_{0})=0.$$

From the shear force balance

(27) $$\int_{0}^{h}\sigma_{xy}d_{z}=\tau_{0}(h_{1}+h_{2}),$$

we obtain

(28) $$\Phi_{1}(x)=-\Phi (x)\frac{G_{12}}{G_{xy}^{\theta }}\frac{1}{f(h)-f(h_{1})},$$

leading to

(29) $$\sigma_{xy}^{\theta }=\tau_{0}^{\theta }+\tau_{0}^{90}\cdot h_{1}\cdot \frac{f^{\prime }(z)}{f(h)-f(h_{1})}\cdot \Phi (x),$$

substituting $\sigma_{xy}^{\theta }$ in the equilibrium equation

(30) $$\frac{\partial \sigma_{yx}}{\partial x}+\frac{\partial \sigma_{yz}}{\partial z}=0,$$

and integrating it with respect to x and applying stress free condition on the surface z = *h* we obtain:

(31) $$\sigma_{yz}^{\theta }=\tau_{0}^{90}\cdot h_{1}\cdot \left[\frac{f(h)-f(z)}{f(h)-f(h_{1})}\right]\cdot \Phi^{\prime }(x).$$

The same equilibrium equation and boundary condition at z = 0 in 90-layer yields

(32) $$\sigma_{yz}^{90}=\tau_{0}^{90}\cdot \Phi^{\prime }(x)\cdot z.$$

It can be checked that the continuity of $\sigma_{yz}$ at the layer interface is satisfied. By introducing a new unknown function φ(z) as

(33) $$\varphi =\left[\frac{f(h)-f(z)}{f(h)-f(h_{1})}\right],$$

the following stress expressions can be written:

(34) $$\begin{array}{l} \sigma_{xy}^{90}=\tau_{0}^{90}\cdot (1-\Phi (x))\\ \sigma_{xy}^{\theta }=\tau_{0}^{\theta }-\tau_{0}^{90}\cdot h_{1}\cdot \Phi (x)\cdot \varphi^{\prime }(z)\\ \sigma_{yz}^{90}=\tau_{0}^{90}\cdot \Phi^{\prime }(x)\cdot z\\ \sigma_{yz}^{\theta }=\tau_{0}^{90}\cdot h_{1}\cdot \varphi (z)\cdot \Phi^{\prime }(x) \end{array}.$$

Finally, the unknown functions φ(z) and Φ(x) have to satisfy boundary conditions:

(35) $$\varphi (h)=0\;\;\;\;\;\varphi (h_{1})=1\;\;\;\;\;\Phi (\pm L_{1})=1.$$

### 4.2. Minimization of Complementary Energy

In the calculation of elastic properties, thermal stress is considered negligible [32], thereby allowing the thermal term to be omitted from the complementary energy expression. Consequently, the complementary energy is equated to the strain energy. The strain energy is associated with the repeating unit situated between two cracks in 90° layer:

(36) $$U^{\theta }=\frac{1}{2}\cdot \int_{-L_{1}}^{L_{1}}\int_{h_{2}}\left[\frac{{\left(\sigma_{xy}^{\theta }\right)}^{2}}{G_{xy}^{\theta }}+\frac{{\left(\sigma_{yz}^{\theta }\right)}^{2}}{G_{yz}^{\theta }}\right]dzdx,$$

(37) $$U^{90}=\frac{1}{2}\cdot \int_{-L_{1}}^{L_{1}}\int_{h1}\left[\frac{{\left(\sigma_{xy}^{90}\right)}^{2}}{G_{12}}+\frac{{\left(\sigma_{yz}^{90}\right)}^{2}}{G_{12}}\right]dzdx.$$

Substituting Equation (34) into Equations (36) and (37) and conducting the Z-integral yields the subsequent expression for the complementary energy of the laminate in the event of a rupture in the 90-layer.

(38) $$U^{LAM}=U^{\theta }+U^{90}=U_{0}^{LAM}+\frac{{\left(\tau_{0}^{90}\right)}^{2}h_{1}}{2G_{12}}\int_{-L_{1}}^{L_{1}}[A_{1}\cdot \Phi^{2}(x)+A_{2}\cdot {h_{1}}^{2}\cdot {\left[\Phi^{\prime }(x)\right]}^{2}]dx,$$

where

(39) $$A_{1}=1+\frac{G_{12}}{G_{xy}^{\theta }}k_{1}\;\;\;\;\;A_{2}=\frac{1}{3}+\frac{G_{12}}{G_{yz}^{\theta }}k_{2},$$

(40) $$k_{1}{=h}_{1}\int_{h_{1}}^{h}[\varphi^{\prime }{\left(z)\right]}^{2}{\mathrm{dz}\;\;\;\;\;\;k}_{1}=\frac{1}{h_{1}}\int_{h_{1}}^{h}[\varphi {\left(z)\right]}^{2}\mathrm{dz}.$$

The function Φ that minimizes (38) has to satisfy the Euler–Lagrange equation

(41) $$\frac{d}{dx}(\frac{\partial L}{\partial \Phi^{\prime }})-\frac{\partial L}{\partial \Phi }=0,$$

which leads to the following equation for Φ(*x*) determination:

(42) $${h_{1}}^{2}\cdot A_{2}\cdot \Phi^{\prime \prime }(x)-A_{1}\cdot \Phi (x)=0.$$

The solution of (42), which satisfies the boundary condition (35), is

(43) $$\Phi =\frac{\cosh(\mu \frac{x}{h_{1}})}{\cosh(\mu \frac{L_{0}}{h_{1}})},\;\mathrm{where}\;\mu =\sqrt{\frac{A_{1}}{A_{2}}}=\sqrt{\frac{1+\frac{G_{12}}{G_{xy}^{\theta }}k_{1}}{\frac{1}{3}+\frac{G_{12}}{G_{yz}^{\theta }}k_{2}}}.$$

The solution provided by Equation (43) yields a minimum value for the complementary energy corresponding to the selected value of φ(z), suggesting that an additional minimization of φ(z) can be attained through a suitable selection. To facilitate this objective, the complementary energy expression (38) can be restructured as follows:

(44) $$U^{LAM}=U_{0}^{LAM}+\frac{{\left(\tau_{0}^{90}\right)}^{2}h_{1}}{2G_{12}}\left\{\int_{-L_{1}}^{+L_{1}}A_{1}\cdot \Phi^{2}(x)dx+A_{2}\cdot d^{2}\cdot [\Phi (x)\cdot \Phi^{\prime }(x){|}_{-L_{1}}^{+L_{1}}-\int_{-L_{1}}^{+L_{1}}\Phi (x)\Phi^{\prime \prime }(x)]dx]\right\}.$$

Replacing $\Phi^{\prime \prime }$ in the second integral via Φ as follows from (42), we obtain

(45) $$U^{LAM}=U_{0}^{LAM}+\frac{{\left(\tau_{0}^{90}\right)}^{2}{h_{1}}^{3}}{2G_{12}}A_{2}2\Phi^{\prime }(L_{1}).$$

Substituting in (45), $A_{2}$ given by (39) and expressing $\Phi^{\prime }(L_{1})$ from (43), we obtain

(46) $$U^{LAM}=U_{0}^{LAM}+\frac{{\left(\tau_{0}^{90}\right)}^{2}h_{1}^{2}\mu }{2G_{12}}(\frac{1}{3}+\frac{G_{12}}{G_{yz}^{\theta }}k_{2})\tan h(\mu \frac{L_{1}}{h_{1}})\;.$$

Selecting different functions φ(z), we will have different values of $K_{2}$ and µ, leading to different values $U^{LAM}$. The lowest value will correspond to the most accurate stress distribution on average.

### 4.3. Shear Modulus Expression

The shear modulus of the damaged laminate can be determined using $u^{LAM}$. The strain energy density of the RVE is calculated as follows:

(47) $$\gamma_{xy}^{LAM}=\frac{\partial \mu^{LAM}}{\partial \tau_{0}}.$$

The strain energy density can be obtained by dividing (46) with the RVE volume. Moreover, the shear stress $\tau_{0}^{90}$ in Equation (46) can be represented by the variable $\tau_{0}$ as per Equation (24), and the strain energy density for undamaged laminates is given by $\tau_{0}^{2}/2G_{xy0}$. Performing differentiation according to (47), we obtain

(48) $$\gamma_{xy}^{LAM}=\frac{\tau_{0}}{G_{xy0}}+\frac{\tau_{0}G_{12}}{(G_{xy0}{)}^{2}}\frac{h_{1}^{2}}{(h_{1}+h_{2})L_{0}}\mu (\frac{1}{3}+\frac{G_{12}}{G_{yz}^{\theta }}k_{2})\tan h(\mu \frac{L_{1}}{h_{1}})\;.$$

Since Hooke’s law for the damaged laminate has to have the form (24), the following shear modulus expression for the damaged laminate can be obtained:

(49) $$\frac{G_{xy}}{G_{xy0}}=\frac{1}{1+\frac{G_{12}}{G_{xy0}}\frac{h_{1}}{\left(h_{1}+h_{2}\right)}\frac{h_{1}}{L_{0}}\mu (\frac{1}{3}+\frac{G_{12}}{G_{yz}^{\theta }}k_{2})\tan h(\mu \frac{L_{1}}{h_{1}})\;}.$$

For cross-ply laminates, $G_{xy}^{\theta }=G_{12},G_{yz}^{\theta }=G_{23}$ and $G_{xy0}=G_{12}$. Then, the expressions for the shear modulus can be re-written as

(50) $$\frac{G_{xy}}{G_{xy0}}=\frac{1}{1+\frac{h_{1}}{\left(h_{1}+h_{2}\right)}\frac{h_{1}}{L_{0}}\mu (\frac{1}{3}+\frac{G_{12}}{G_{23}}k_{2})\tan h(\mu \frac{L_{1}}{h_{1}})\;},\;\mu =\sqrt{\frac{1+k_{1}}{\frac{1}{3}+\frac{G_{12}}{G_{23}}k_{2}}}.$$

As the distance from the crack tip (layer interface) increases, it is anticipated that the shear stress disturbance will decay, necessitating a decrease in the absolute value of the function φ(z) and its first derivative. Natural selection exhibiting such characteristics is described by an exponential function. The shape function incorporates a shape parameter n, which serves to differentiate the minimum value of the complementary energy specified by Equation (46). For the present study, the function that satisfies boundary conditions (35) is selected in the following form:

(51) $$\varphi (z)=\frac{1}{e^{\frac{n\cdot h_{2}}{h_{1}}}-1}\cdot \left(e^{\frac{n\cdot (z-h)}{h_{1}}}-1\right)\;\mathrm{and}\;\varphi^{\prime }=-\frac{n}{h_{1}}\frac{e^{\frac{n\cdot (z-h)}{h_{1}}}}{e^{\frac{n\cdot h_{2}}{h_{1}}}-1},$$

where n is the number of single-layer plates of 0° layer, e is the natural base number, and z is the thickness coordinate.

Using (18), the parameters $k_{1}$, $k_{2}$ are calculated as follows:

(52) $$k_{1}=\frac{n}{2}\frac{e^{\frac{n\cdot h_{2}}{h_{1}}}+1}{e^{\frac{n\cdot h_{2}}{h_{1}}}-1},\;k_{2}=\frac{\frac{h_{2}}{h_{1}}-\frac{1}{2n}\left(1-e^{\frac{2n\cdot h_{2}}{h_{1}}}\right)+\frac{2}{n}\left(1-e^{\frac{n\cdot h_{2}}{h_{1}}}\right)}{{\left(e^{\frac{n\cdot h_{2}}{h_{1}}}-1\right)}^{2}}.$$

## 5. Validation of Model Feasibility

**Figure 2:**
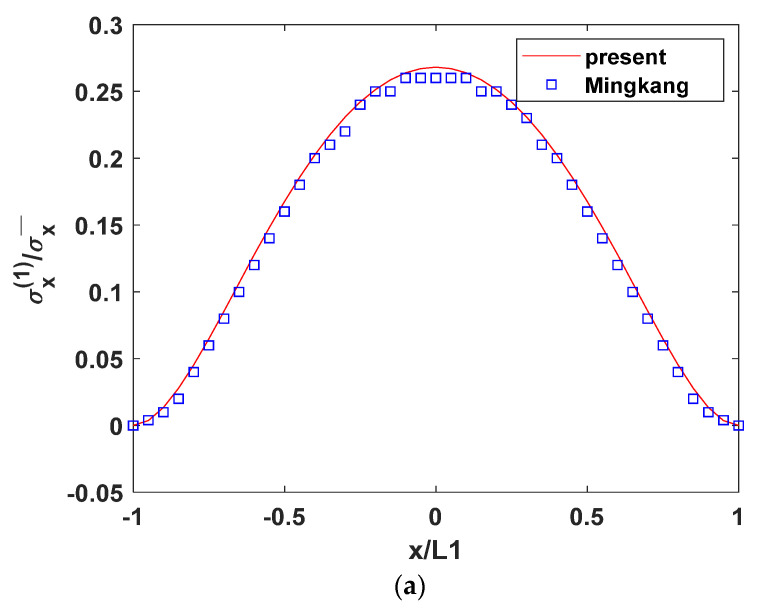

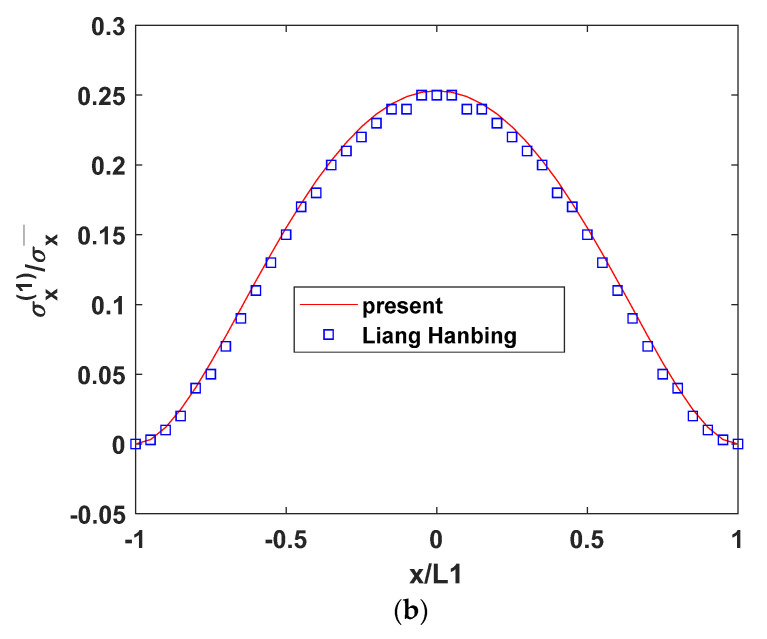
(**a**) Distribution of normal stress $\sigma_{x}^{\left(1\right)}$ in 90° layer of the laminated plate ${\left[30/90_{3}\right]}_{s}$ with open crack. (**b**) Distribution of normal stress $\sigma_{x}^{\left(1\right)}$ in 90° layer of laminated plate ${\left[30_{2}/90_{2}\right]}_{s}$ with open crack. The data points of the blue box (**a** and **b**) come from [8] and [16] respectively.

Given that we are examining smooth open cracks, $K_{1}$ and $K_{2}$ are both equal to zero. Based on Equations (1) and (2), it can be inferred that $\lambda_{\sigma }=0$ and $\lambda_{\tau }=0$. To validate the accuracy of the variational model, analyses were conducted on laminates made from glass/epoxy resin 1 (or glass/epoxy resin 2). At a crack density of CD = 15 (cr/cm), the distribution of normal and shear stresses within the 90° layer was calculated using MATLAB 2018a software, as depicted in Figure 2a,b. In this paper, the normal stress of the 90° smooth crack layer is compared with the calculated data of Mingkang [8] and Liang Hanbing [16]. The basic theory of composite material mechanics and the energy principle are used in this paper to build the model, which is slightly different from the above references; however, in some aspects, the concrete verification process is used to consider the interface parameter Kt of the non-ideal crack in Mingkang [8]. However, this topic is to consider the smooth crack condition, so that Kt = 10 will degenerate into a smooth surface crack to discuss together, while the non-thickness spring model in this paper when considering the smooth crack condition, $K_{1}$ and $K_{2}$ are zero, can also degenerate into a smooth crack, and the simulation results of Liang Hanbing [16] are basically consistent. The results show that the model has higher precision, the modeling process is more concise, and the efficiency of the calculation results is improved.

## 6. Stress Distribution Analysis

### 6.1. Influence of Fiber Laying Angle on Stress Distribution of Laminates with Matrix Cracks

To examine the impact of fiber layering angle on the mechanical properties of symmetric laminates with opening crack damage when subjected to tensile loading, a set of glass/epoxy resin 2 laminates ${\left[\theta /90_{2}\right]}_{s}$ were numerically analyzed, and the material constants were derived from Mingkang [8]: $E_{1}=45.0\;GPa$, $E_{2}=15.0\;GPa$, $G_{12}=5.00\;GPa$, $G_{23}=5.36\;Gpa$, $\mu_{12}=0.30$, $\mu_{23}=0.40$, and a thickness of $t_{0}\;is\;0.100\;mm$ for each lamina. The crack density was set at 0.6 (cr/mm).

**Figure 3:**
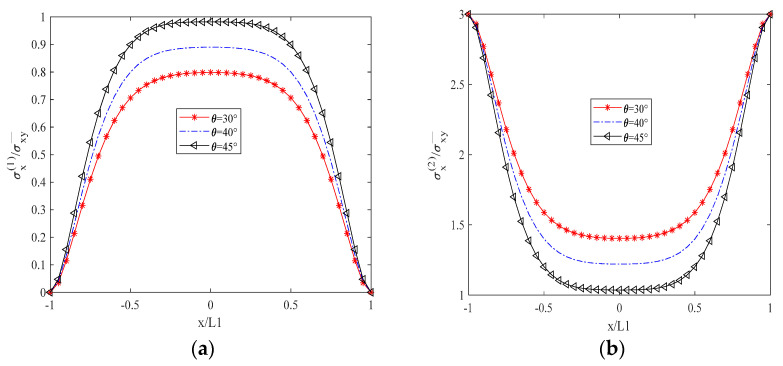
Distribution of Normal Stress in the X–Direction for Two Layers in a Unit Element. (**a**) Layer at 90° Angle. (**b**) Layer at θ° Angle.

**Figure 4:**
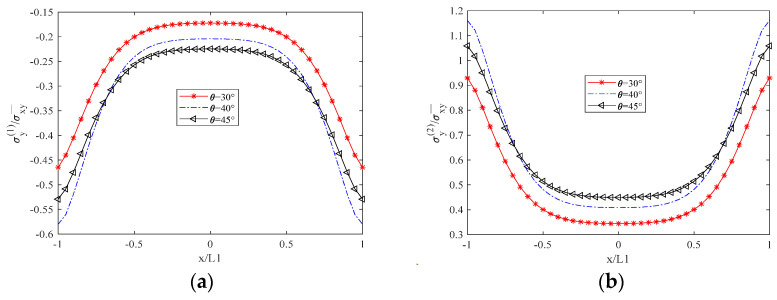
Distribution of Normal Stress in the Y–Direction for Two Layers in a Unit Element. (**a**) Layer at 90° Angle. (**b**) Layer at θ° Angle.

**Figure 5:**
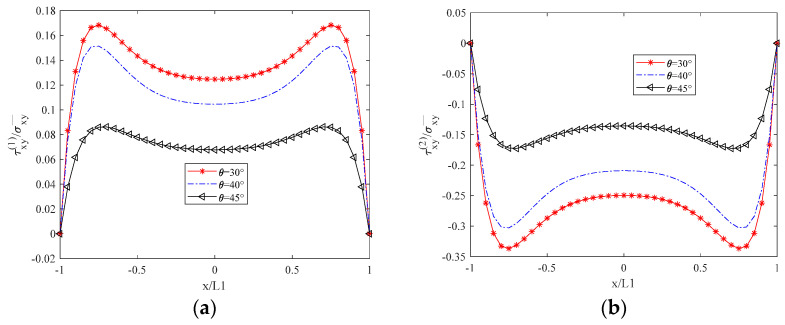
Shear Stress Distribution in the XY Plane for Two Paving Layers Within a Unit Element. (**a**) Layer at 90° Angle. (**b**) Layer at θ° Angle.

Figure 3 illustrates that $\sigma_{x}^{\left(1\right)}$, the normal stress in the x-direction of the 90° layer, is zero at the unit boundary (crack surface), as dictated by the boundary conditions. $\sigma_{x}^{\left(2\right)}$ represents the normal stress perpendicular to the fiber direction in the θ-degree layer, decreasing as the θ angle increases due to the reduced alignment of the fiber direction with the x-axis. The distribution of $\sigma_{x}^{\left(2\right)}$ is inversely proportional to that of σx1, with $\sigma_{x}^{\left(2\right)}$ being significantly greater in magnitude, signifying that the θ-degree layer is the primary load-bearing layer. Conversely, the load-bearing capacity of the 90-degree layer is substantially diminished following cracking. As depicted in Figure 4, $\sigma_{y}^{\left(1\right)}$ represents the normal stress in the y-direction of the 90° cracked layer. Its distribution shape is opposite to that of $\sigma_{y}^{\left(2\right)}$, and its magnitude is lower than that of $\sigma_{y}^{\left(1\right)}$, which further indicates that the crack diminishes the bearing capacity. $\sigma_{y}^{\left(2\right)}$ denotes the normal stress parallel to the fiber direction, and its magnitude increases with an increasing θ angle. This behavior is in contrast to the decrease in $\sigma_{x}^{\left(1\right)}$ with increasing θ angle. In Figure 5, $\tau_{xy}^{\left(1\right)}$ and $\tau_{xy}^{\left(2\right)}$ represent the shear stresses within the xy plane of the 90° cracked layer and the θ° layer, respectively, with their distributions and shapes being mutually inverse. Due to the presence of the crack surface, the boundary values are zero, with $\tau_{xy}^{\left(2\right)}$ exceeding $\tau_{xy}^{\left(1\right)}$ in magnitude. The distribution of both normal and shear stresses within the plane is independent of the material thickness, and this distribution remains consistent across any thickness interface.

### 6.2. Influence of Material Properties on Stress Distribution of Laminates with Matrix Cracks

The present study is designed to investigate the influence of various material parameters on the behavior of damaged laminates, utilizing glass epoxy resin and carbon fiber as the selected materials. The material parameters are derived from the articles of Hashin [30]. Table 1 presents the material constants for two types of epoxy resin and carbon fiber:

The variation in surface stress of laminate ${\left[30_{2}/90_{2}\right]}_{s}$ with material parameters is discussed in the case of open matrix crack.

**Figure 6:**
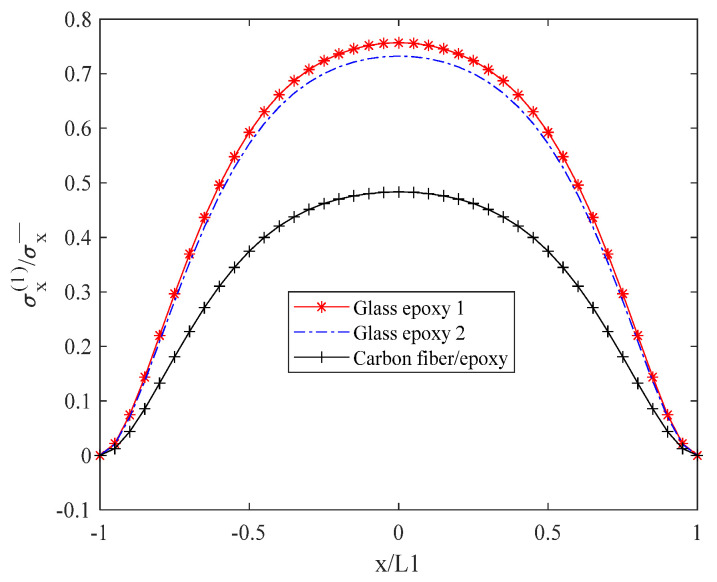
Normal stress $\sigma_{x}^{\left(1\right)}$ distribution of laminates ${\left[30_{2}/90_{2}\right]}_{s}$ with open cracks.

Figure 6 illustrates that the internal normal stress $\sigma_{x}^{\left(1\right)}$ exhibits a descending order from glass/epoxy resin 1 to glass/epoxy resin 2, and finally to carbon fiber/epoxy resin. Moreover, at *x* = 0, the stress values are maximized for both materials.

**Figure 7:**
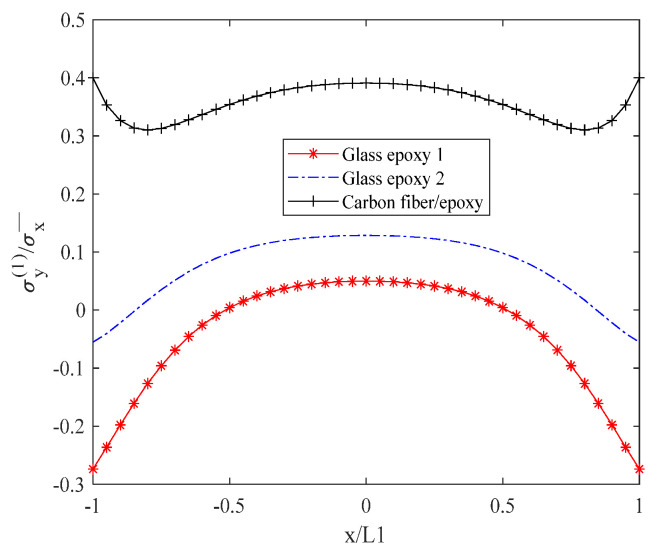
Normal stress $\sigma_{y}^{\left(1\right)}$ distribution of laminates ${\left[30_{2}/90_{2}\right]}_{s}$ with open cracks.

Figure 7 demonstrates the in-plane normal stress $\sigma_{y}^{\left(1\right)}$ in descending order, with the carbon fiber/epoxy resin exhibiting the highest stress, followed by glass/epoxy resin 2, and then glass/epoxy resin 1. For the carbon fiber/epoxy resin, the stress value increases gradually within the range of −0.8 $L_{1}$ to 0 $L_{1}$ (inclusive of 0.8 $L_{1}$ to 0 $L_{1}$), reaching a maximum of approximately 0.4. For glass/epoxy resin 2, the stress value remains stable at approximately 0.1 within the interval of −0.6 $L_{1}$ to 0 $L_{1}$ (inclusive of 0.6 $L_{1}$ to 0 $L_{1}$). For glass/epoxy resin 1, the stress value is stable at about 0.05 within the range of −0.4 $L_{1}$ to 0.41 $L_{1}$.

**Figure 8:**
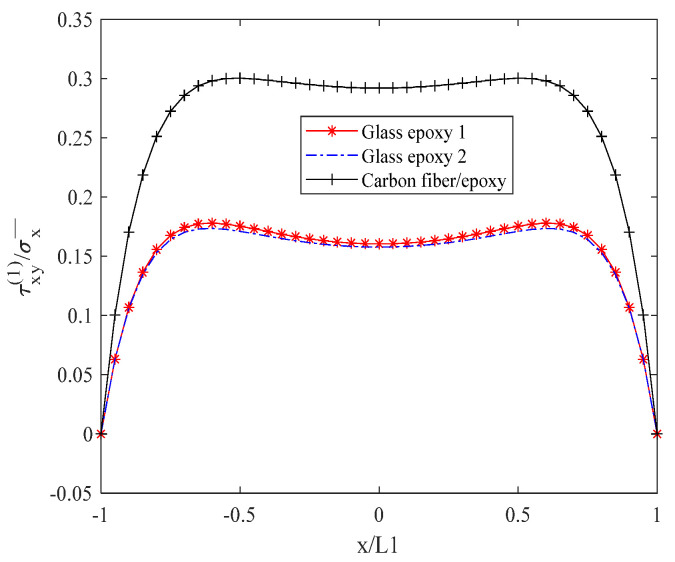
Normal stress $\tau_{xy}^{\left(1\right)}$ distribution of laminates ${\left[30_{2}/90_{2}\right]}_{s}$ with open cracks.

Figure 8 illustrates the in-plane shear stress $\tau_{xy}^{\left(1\right)}$ in a descending order, with the carbon fiber/epoxy resin demonstrating the highest stress, followed by glass/epoxy resin 1, and then glass/epoxy resin 2. Within the interval of −0.55 $L_{1}$ to 0.55 $L_{1}$, the maximum shear stress value for the carbon fiber/epoxy resin remains stable at approximately 0.292. For glass/epoxy resin 1, the maximum shear stress value is stable at approximately 0.1605, while for glass/epoxy resin 2, it is stable at approximately 0.1578. The subsequent discussion examines the variation in interlayer shear stress in relation to material parameters.

**Figure 9:**
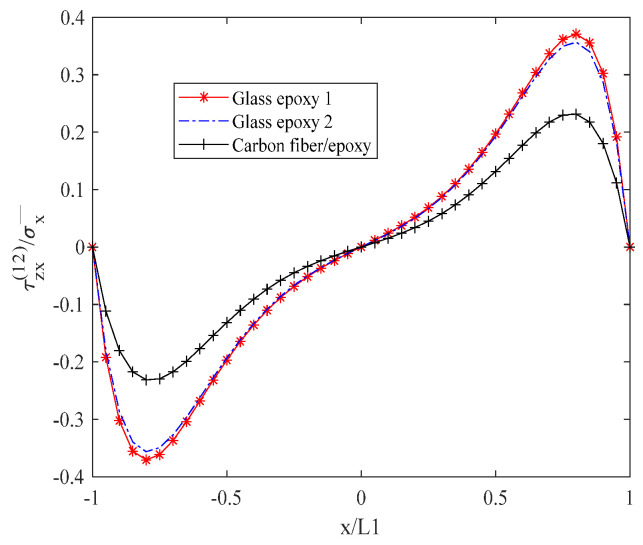
Shear stress $\tau_{\mathrm{zx}}^{\left(12\right)}$ of laminates ${\left[30_{2}/90_{2}\right]}_{s}$ with open cracks at the interface.

Figure 9 depicts the interlayer shear stress $\tau_{\mathrm{zx}}^{\left(12\right)}$ in a descending order, with glass/epoxy resin 1 exhibiting the highest stress, followed by glass/epoxy resin 2, and carbon fiber/epoxy resin. Additionally, the stress value is zero at x = 0, and it exhibits an increasing trend followed by a decreasing trend within the range of 0 to 1.

**Figure 10:**
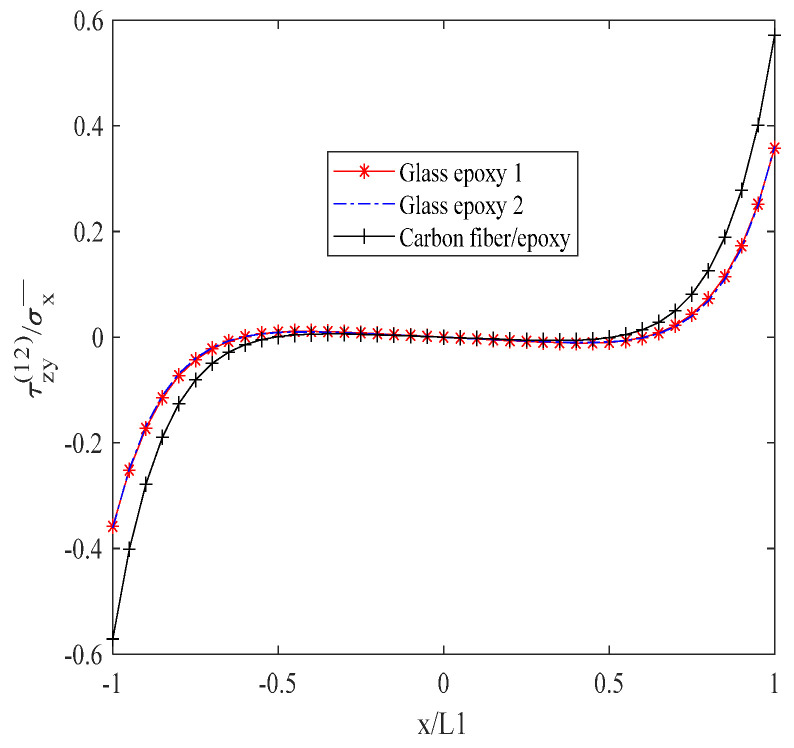
Shear stress $\tau_{zy}^{\left(12\right)}$ of laminates ${\left[30_{2}/90_{2}\right]}_{s}$ with open cracks at the interface.

Figure 10 reveals the interlayer shear stress $\tau_{zy}^{\left(12\right)}$ in a descending order, with the carbon fiber/epoxy resin exhibiting the highest stress, followed by glass/epoxy resin 2, and then glass/epoxy resin 1. Within the interval of −0.6 $L_{1}$ to 0.6 $L_{1}$, the stress value remains constant at zero. Between x = 0.6 $L_{1}$ and 0.8 $L_{1}$, the stress value exhibits a gradual increase. Between x = −0.6 $L_{1}$ and −0.8 $L_{1}$, the stress value undergoes a gradual decrease.

**Figure 11:**
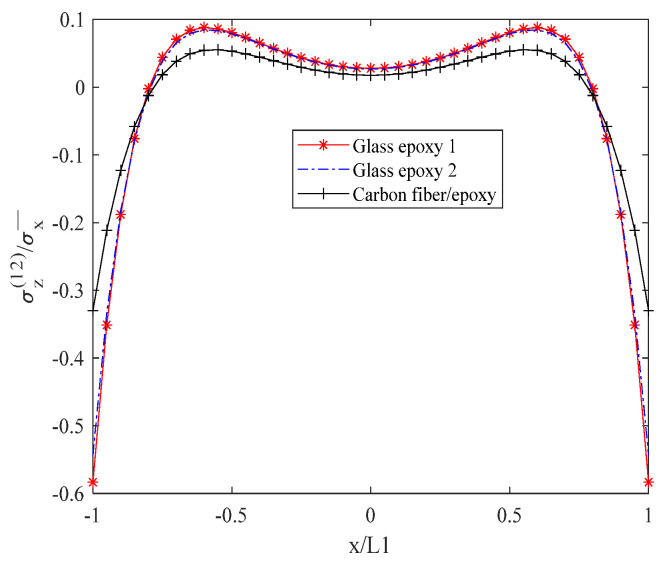
Normal stress $\sigma_{z}^{\left(12\right)}$ of laminates ${\left[30_{2}/90_{2}\right]}_{s}$ with open cracks at the interface.

Figure 11 shows the order of normal stress $\sigma_{z}^{\left(12\right)}$ between layers from large to small: carbon fiber/epoxy resin, glass/epoxy resin 1, and glass/epoxy resin 2. When x is between −0.5 $L_{1}$ and 0.5 $L_{1}$, the stress value reaches its peak, between 0 and 0.1.

## 7. Shear Modulus Evolution

To advance the investigation into the degradation of stiffness in composite laminates resulting from crack damage, orthogonal laminates ${\left[0_{n}/90_{2}\right]}_{s}$ composed of glass/epoxy resin 1 and glass/epoxy resin 2 were selected, respectively, with their material properties presented in Table 1. The variable n represents the number of single layers of 0° layers.

**Figure 12:**
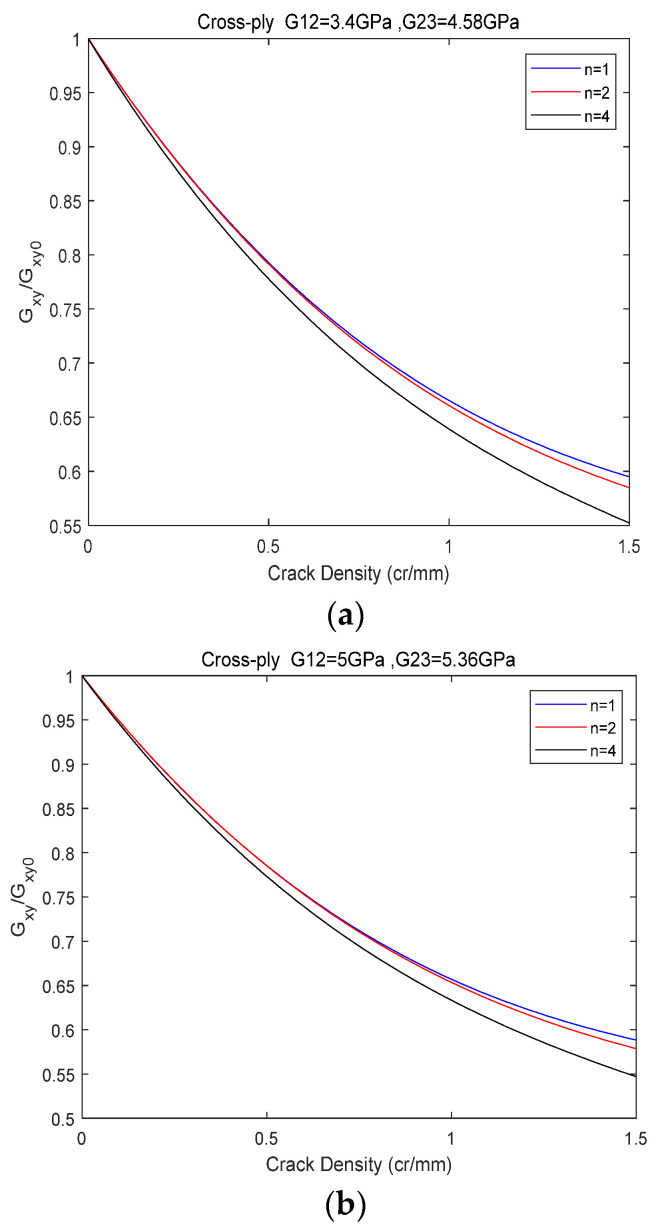
Shear modulus evolution of orthogonal laminates ${\left[0_{n}/90_{2}\right]}_{s}$ with matrix cracks. (**a**) Glass/epoxy 1. (**b**) Glass/epoxy 2.

**Figure 13:**
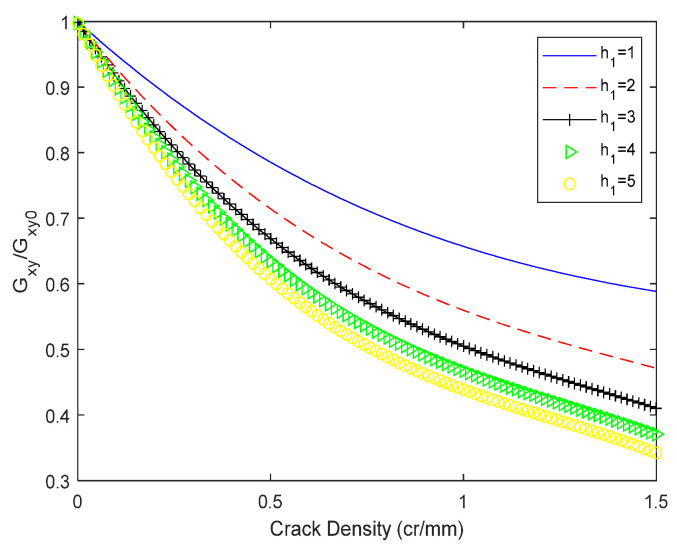
Effect of 90° thickness on the shear modulus of a cross-ply laminate ${\left[0/90_{2}\right]}_{s}$. The thickness of the 90-layer is $h_{1}$ ∈ [1, 5] mm, and the 0-layer thickness is 1 mm.

**Figure 14:**
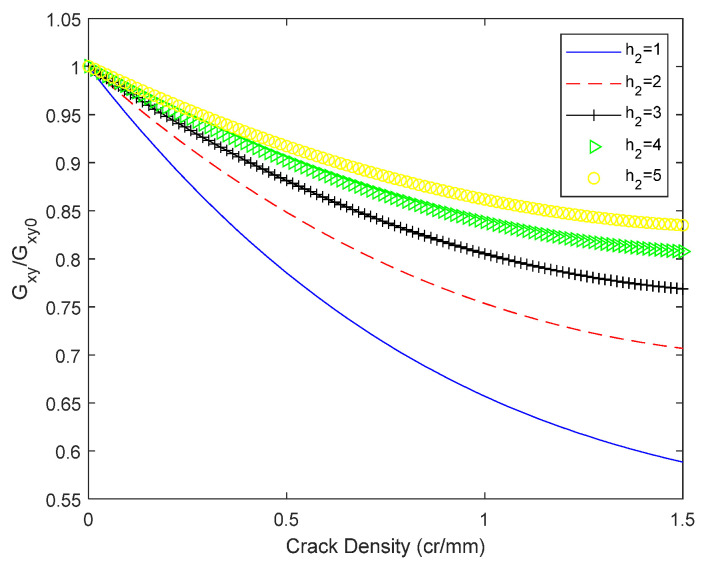
Effect of 90° thickness on the shear modulus of a cross-ply laminate ${\left[0/90_{2}\right]}_{s}$. The thickness of the 0-layer is $h_{2}$ ∈ [1, 5] mm, and the 90-layer thickness is 1 mm.

Figure 12 illustrates that for both glass/epoxy resin 1 and glass/epoxy resin 2, the shear modulus exhibits a rapid decline as the crack density (CD) increases. Moreover, this decreasing trend is more pronounced with an increasing number of layers in the 0° orientation. These findings suggest a direct correlation between the structural configuration of the laminates and their resistance to crack propagation. In Figure 13, The thickness of the outermost 0° layer is maintained constant at 1 mm, whereas the thickness of the 90° layer varies, yielding distinct values. Specifically, thicknesses ranging from 1 to 5 mm were examined, with an incremental step for the 90° layer thickness increase set at 1 mm. In this analysis, the values for both in-plane and out-of-plane shear moduli are maintained constant, with $G_{12}$ = 4.58 GPa and $G_{23}$ = 3.4 GPa. An inverse relationship is observed between layer thickness and the reduction of $G_{xy}$, with greater thickness correlating with increased reduction. The findings indicate that the thickness of the transverse layer influences the slope of the shear modulus $G_{xy}$ curve. Additionally, it is concluded that crack density saturation occurs at an earlier stage with increasing layer thickness. An identical method was employed to quantify the impact of the thickness of the 0° layer. The 90° layer’s thickness was kept constant at 1 mm, while the 0° layer’s thickness was assigned values ranging from 1 to 5 mm, increasing in 1 mm increments. The results are presented in Figure 14. Figure 14 shows the significance of the longitudinal plies on the final shear properties of the cross-ply. This becomes evident as the reduction in shear modulus due to cracking at saturation reaches nearly 16% for 5 mm thick layers of 0, whereas it approaches 42% for 1 mm thick layers (i.e., a single layer of 0).

## 8. Conclusions

This manuscript is structured into two principal sections. The initial section delves into the stress distribution model of smooth cracked symmetric composite laminates, examining variations in fiber orientation and material properties within the framework of the variational method and the principle of minimum potential energy. Adhering to the principle of minimum complementary energy, the paper derives the governing equations through variable differentiation and establishes the solution constants and undetermined coefficients in accordance with the boundary conditions. This culminates in the determination of stress components across each laminate layer. To validate the accuracy of the developed simulation model, a comparative analysis was conducted using simulation data provided by Ming Kang [7] and Liang Hanbing [16]. In subsequent chapters, the paper employs the complementary energy minimization theory to forecast the degradation of the shear modulus in composite laminates under varying structural configurations. The findings of this research have significantly advanced the understanding of the mechanical properties of composite laminates with matrix cracks. They offer theoretical insights into how different structural parameters, external loads, and material properties influence mechanical properties, thereby contributing to the theoretical foundation and practical application of composite mechanics.

(1) In the instance of smooth open cracks, the alteration in the fiber orientation within θ layer significantly affects the internal positive (tangential) stress distribution. Due to the variation in the fiber orientation angle, the load component in the x (or y) direction corresponding to the fiber orientation also changes. Such variations result in distinct stress distributions across the layers. The specific manifestations are as follows. For in-plane stress $\sigma_{x}$, the stress value in the cracked layer increases concomitantly with the increase in the fiber orientation angle. When the spatial horizontal coordinate x is between −0.5 $L_{1}$ and 0.5 $L_{1}$, the in-plane normal stress $\sigma_{x}^{\left(1\right)}$ reaches its peak value. However, the stress value in the θ layer exhibits an opposite trend, reaching its minimum value within the same coordinate range. Similarly, for the in-plane normal stress $\sigma_{y}$, the stress value in the cracked layer decreases as the fiber orientation angle increases. When the spatial horizontal coordinate x is between −0.5 $L_{1}$ and 0.5 $L_{1}$, the in-plane normal stress $\sigma_{y}^{\left(1\right)}$ attains its peak value. Conversely, the θ layer exhibits an opposing trend, with stress values reaching their minimum within the same coordinate range. Concerning in-plane shear stress $\tau_{xy}$, the stress value in the cracked layer decreases as the fiber orientation angle increases. When the spatial horizontal coordinate x is at −0.3 $L_{1}$ and 0.3 $L_{1}$, the in-plane shear stress $\tau_{xy}^{\left(1\right)}$ reaches its peak value. Conversely, the stress value in θ layer follows an inverse pattern, achieving its lowest value at these coordinates. It can be observed that the bearing capacity of the cracked layer is greatly reduced due to the presence of matrix cracks, with the external load being mainly carried by the θ layer.

(2) For the same opening crack, variations in material parameters significantly influence both the in-plane and interlayer positive (tangential) stress distributions, with the impact varying depending on the stress type and the specific material parameter. For in-plane stress, the in-plane normal stress $\sigma_{x}^{\left(1\right)}$ attains its peak when the horizontal coordinate x falls within the range of −0.2 $L_{1}$ to 0.2 $L_{1}$. For in-plane shear stress $\tau_{xy}^{\left(1\right)}$, the stress reaches its peak when the spatial horizontal coordinate x is between −0.6 $L_{1}$ and 0.6 $L_{1}$. The normal stress $\sigma_{y}^{\left(1\right)}$ in the in-plane does not follow a definitive pattern. However, stress variation patterns differ based on varying fiber orientation angles. For interlaminar stress, when the horizontal coordinate x lies within the range of 0 $L_{1}$ to 1 $L_{1}$ (or 0 $L_{1}$ to −1 $L_{1}$), the interlaminar shear stress $\tau_{\mathrm{zx}}^{\left(12\right)}$ initially peaks before declining to zero. Within the horizontal coordinate range of 0.8 $L_{1}$ to 1 $L_{1}$ (or −0.8 $L_{1}$ to −1 $L_{1}$), the interlayer shear stress $\tau_{zy}^{\left(12\right)}$ gradually increases, culminating at its peak value. The interlaminar shear stress $\sigma_{z}^{\left(12\right)}$ attains its peak within the horizontal coordinate range of −0.6 $L_{1}$ to 0.6 $L_{1}$. In the horizontal coordinate range from 0.5 $L_{1}$ to 1 $L_{1}$ (or 0.5 $L_{1}$ to −1 $L_{1}$), the interlayer shear stress progressively diminishes.

(3) Matrix cracking not only influences the stress distribution within composite laminates but also significantly impacts the evolution of their stiffness. For instance, variations in structural parameters, including the number of single-layer plates and the thickness of the 0° layer, are introduced. Moreover, this decreasing trend is more pronounced with an increasing number of layers in the 0° orientation. These findings suggest a direct correlation between the structural configuration of the laminates and their resistance to crack propagation. With the outermost 0° layer thickness maintained at 1 mm, the impact of transverse layer thickness on the shear modulus is investigated by varying the 90° cracked layer thickness from 1 to 5 mm in 1 mm increments. The findings indicate that the transverse layer thickness significantly influences the slope of the shear modulus curve. An increase in layer thickness is associated with a decrease in the slope of the shear modulus curve. This is primarily due to the increased proportion of the cracked layer within the laminate, which corresponds to a deeper matrix crack and a more rapid natural decline in the shear modulus. An identical methodology was employed to quantify the impact of 0° layer thickness. The 90° cracked layer thickness is maintained at 1 mm, with the 0° layer thickness varying from 1 to 5 mm in 1 mm increments. The slope of the shear modulus curve’s decline for the 0° layer is inverted relative to that of the cracked layer. At saturation, a 5 mm thick layer 0 exhibits a shear modulus reduction of approximately 16% due to cracking, whereas a 1 mm thick layer (i.e., a single layer 0) experiences a reduction of nearly 42%. 

## Figures and Tables

**Table 1:** Properties of unidirectional materials.

	$E_{1}$	$E_{2}$	$G_{12}$	$G_{23}$	$\upsilon_{12}$	$\upsilon_{23}$
glass/epoxy 1	41.7 Gpa	13.0 GPa	3.40	4.58	0.3	0.42
glass/epoxy 2	45.0 Gpa	15.0 Gpa	5.00	5.36	0.30	0.40
carbon fiber/epoxy resin	148	9.57	4.5	4.2	0.356	0.49

## Data Availability

Data are contained within the article.

## Appendix A

$$\begin{array}{l} A_{11}=[\frac{1}{4}S_{33}^{\left(1\right)}(h^{2}t_{1}-\frac{2}{3}ht_{1}^{2}+\frac{1}{5}t_{1}^{3})+\frac{1}{20}S_{33}^{\left(2\right)}t_{2}^{3}]\frac{1}{S_{11}^{\left(1\right)}L_{1}^{3}}\\ B_{11}=-\frac{1}{3}[S_{55}^{\left(1\right)}t_{1}+S_{13}^{\left(1\right)}(3h-t_{1})+S_{55}^{\left(2\right)}t_{2}-S_{13}^{\left(2\right)}t_{2}]\frac{1}{S_{11}^{\left(1\right)}L_{1}}\\ B_{12}=B_{21}=\frac{1}{6}(S_{36}^{\left(2\right)}-2S_{45}^{\left(2\right)})\frac{t_{2}}{S_{11}^{\left(1\right)}L_{1}}\\ B_{13}=B_{31}=\frac{1}{6}[S_{23}^{\left(1\right)}(3h-t_{1})-S_{23}^{\left(2\right)}t_{2}]\frac{1}{S_{11}^{\left(1\right)}L_{1}}\\ B_{22}=-\frac{S_{44}^{\left(1\right)}t_{1}+S_{44}^{\left(2\right)}t_{2}}{3S_{11}^{\left(1\right)}L_{1}}\\ C_{11}=(\frac{1}{t_{1}}+\frac{S_{11}^{\left(2\right)}}{S_{11}^{\left(1\right)}t_{2}})L_{1}\\ C_{12}=C_{21}=\frac{S_{16}^{\left(2\right)}L_{1}}{S_{11}^{\left(1\right)}t_{2}}\\ C_{13}=C_{31}=-(S_{12}^{\left(1\right)}\frac{1}{t_{1}}+S_{12}^{\left(2\right)}\frac{1}{t_{2}})\frac{L_{1}}{S_{11}^{\left(1\right)}}\\ C_{22}=(S_{66}^{\left(1\right)}\frac{1}{t_{1}}+S_{66}^{\left(2\right)}\frac{1}{t_{2}})\frac{L_{1}}{S_{11}^{\left(1\right)}}\\ C_{23}=C_{32}=-\frac{S_{26}^{\left(2\right)}L_{1}}{S_{11}^{\left(1\right)}t_{2}}\\ C_{33}=(S_{22}^{\left(1\right)}\frac{1}{t_{1}}+S_{22}^{\left(2\right)}\frac{1}{t_{1}})\frac{L_{1}}{S_{11}^{\left(1\right)}}\\ D_{1}=-S_{16}^{\left(2\right)}\frac{h}{t_{2}}\frac{1}{S_{11}^{\left(1\right)}}\\ D_{2}=-S_{66}^{\left(2\right)}\frac{1}{S_{11}^{\left(1\right)}}\frac{h}{t_{2}}\\ D_{3}=-S_{16}^{\left(2\right)}\frac{h}{t_{2}}\frac{1}{S_{11}^{\left(1\right)}} \end{array}$$

where $S_{ij}^{\left(k\right)}(i,j=12,\ldots ,6;k=1,2)$ are compliance elements in different layers for the Cartesian coordinate system (Oxyz).

## Appendix B

$$\begin{array}{l} a_{11}=A_{11}-\frac{B_{13}^{2}}{C_{33}}\\ b_{11}=B_{11}-2\frac{B_{13}C_{31}}{C_{33}}\\ b_{12}=B_{12}-B_{13}\frac{C_{32}}{C_{33}}\\ b_{21}=b_{12}\\ b_{22}=B_{22}\\ c_{11}=C_{11}-\frac{C_{13}^{2}}{C_{33}}\\ c_{12}=C_{12}-C_{13}\frac{C_{32}}{C_{33}}\\ c_{21}=c_{12}\\ c_{22}=C_{22}-C_{23}\frac{C_{32}}{C_{33}}\\ \Lambda_{1}=\lambda_{1}-C_{13}\frac{\lambda_{3}}{C_{33}}\\ \Lambda_{2}=\lambda_{2}-C_{23}\frac{\lambda_{3}}{C_{33}}\\ d_{1}=D_{1}-C_{13}\frac{D_{3}}{C_{33}}\\ d_{2}=D_{2}-C_{23}\frac{D_{3}}{C_{33}} \end{array}$$
